# Clinical guidelines for postpartum women and infants in primary care–a systematic review

**DOI:** 10.1186/1471-2393-14-51

**Published:** 2014-01-29

**Authors:** Crishan Haran, Mieke van Driel, Benjamin L Mitchell, Wendy E Brodribb

**Affiliations:** 1 Discipline of General Practice, School of Medicine, The University of Queensland, Royal Brisbane and Women’s Hospital, Level 8, Health Sciences Building, Herston 4029, Australia

**Keywords:** Postpartum care, Clinical guidelines, AGREE II, Maternal health, Infant health

## Abstract

**Background:**

While many women and infants have an uneventful course during the postpartum period, others experience significant morbidity. Effective postpartum care in the community can prevent short, medium and long-term consequences of unrecognised and poorly managed problems. The use of rigorously developed, evidence-based guidelines has the potential to improve patient care, impact on policy and ensure consistency of care across health sectors. This study aims to compare the scope and content, and assess the quality of clinical guidelines about routine postpartum care in primary care.

**Methods:**

PubMed, the National Guideline Clearing House, Google, Google Scholar and relevant college websites were searched for relevant guidelines. All guidelines regarding routine postpartum care published in English between 2002 and 2012 were considered and screened using explicit selection criteria. The scope and recommendations contained in the guidelines were compared and the quality of the guidelines was independently assessed by two authors using the AGREE II instrument.

**Results:**

Six guidelines from Australia (2), the United Kingdom (UK) (3) and the United States of America (USA) (1), were included. The scope of the guidelines varied greatly. However, guideline recommendations were generally consistent except for the use of the Edinburgh Postnatal Depression Scale for mood disorder screening and the suggested time of routine visits. Some recommendations lacked evidence to support them, and levels or grades of evidence varied between guidelines. The quality of most guidelines was adequate. Of the six AGREE II domains, applicability and editorial independence scored the lowest, and scope, purpose and clarity of presentation scored the highest.

**Conclusions:**

Only one guideline provided comprehensive recommendations for the care of postpartum women and their infants. As well as considering the need for region specific guidelines, further research is needed to strengthen the evidence supporting recommendations made within guidelines. Further improvement in the editorial independence and applicability domains of the AGREE ll criteria would strengthen the quality of the guidelines.

## Background

### Introduction

Childbirth and the subsequent postpartum period is an exciting and special life experience for many women. However, it is also a time of great change, physically, mentally and socially for mothers, infants and families. While many mothers and infants transition through this time uneventfully, others find it overwhelming or develop significant health issues that may persist for weeks and months after giving birth. For example, up to 50% of women report tiredness [1-6] and backache [1-7], while a significant proportion describe headaches [1,3-5,7], perineal [2,3,5,6,8] and caesarean wound pain [8]. Many women experience problems such as breast engorgement, sore nipples, mastitis, postpartum anxiety, prolonged bleeding and urinary tract infections [6,9].

Postpartum depression is also common [1-3,6,10] and is often associated with physical and relationship problems [10]. Women with postpartum depression are less likely to attend routine postpartum consultations [11], or to vaccinate their children in a timely manner [12]. The infants of women with depression are more likely to be unsettled [13] and to have delayed language and behaviour problems at three years [14].

Women who rate their health as low are more likely to report symptoms that affect general physical functioning and well-being such as tiredness, headache, musculoskeletal problems, mastitis, perineal pain and dysuria [5].

Overall, nearly 70% of women describe at least one physical problem within the first 12 months postpartum [15]. For 25% of these women the problem is deemed to be of moderate severity and 20% have severe problems [15]. As the presence and severity of postpartum problems increase, there is a corresponding increase in women’s functional limitations including their ability to work, look after children or undertake household tasks, and an increase of depressive symptoms [15].

Some infants also experience problems with reports of hypernatraemic dehydration [16], feeding difficulties [17] and hospital readmission [18,19] in the early days following hospital discharge. Prolonged crying is also one of the most common reasons for seeking medical care during this period [13] with unsettled behaviour being associated with high health service utilization [20].

Although childbirth must be considered a ‘normal’ part of life, considering the significant life changes associated with it and the burden of morbidity in this population, the aim of care in the postpartum period beyond the immediate peripartum phase must be ‘*to detect health problems of mother and/or baby at an early stage, to encourage breastfeeding and to give families a good start*’ [21]. As most women return to the community within a few days of birthing, postpartum care is best delivered in a primary care setting. Therefore it is important that postpartum care is integrated into primary health care. However, there appears to be inconsistency about the timing and content of routine care for mothers and infants in this setting both between and within countries [21-25].

Although there are some concerns about the practicality of clinical practice guidelines [26], they provide evidence-based recommendations to improve health care and outcomes for patients [27]. In the context of the primary care of women and infants following childbirth, evidence-based guidelines have the potential to enhance care and reduce medium and long-term morbidity. As well as offering advice for management in a clinical situation, guidelines may also have an impact at a policy level, helping to ensure consistent care across health care sectors and professions [27]. On the other hand implementation of guidelines in clinical practice requires a comprehensive approach including local policies and contextual issues [28].

The aim of this study was to identify clinical guidelines that address routine postpartum care in the primary care setting, to compare their scope and content, and assess their quality.

## Methods

### Selection of guidelines

PubMed was searched in December 2012 for articles published in the previous 10 years using the terms perinatal, puerperium, postnatal, postpartum and limited by ‘clinical guidelines’. Since guidelines are rarely published in medical journals, a wider search employing guideline specific databases such as the National Guideline Clearinghouse (NGC) and National Institute for Health and Care Excellence (NICE) was undertaken and relevant college websites in English-speaking countries including the Royal College of Obstetricians and Gynaecologists (RCOG), Royal Australian College of General Practitioners (RACGP) and the American Academy of Pediatrics (AAP) were searched for guidelines. Reference lists of identified guidelines were also searched.

To be included in this study the guidelines had to: include recommendations on routine postpartum care and complications arising in the postpartum period; target primary health care providers; aim at a state or nation-wide level; outline recommendations for care with directly cited levels or grades of evidence; include a reference list; and be available in English full text on the internet. No other inclusion and exclusion criteria were used.

### Comparison of guidelines

Each guideline was summarized by one author (CH) to identify key points and recommendations. These summaries were checked by other authors for completeness and accuracy. Direct comparisons of the scope of the guidelines within four themes (maternal health, maternal mental health, infant health and breastfeeding) were tabulated by one author (CH) and then checked by other authors to ensure they were correct. These broad themes were chosen to assist with comparisons between guidelines and to cover the majority of the recommendations within the all guidelines.

For comparison of the content of the recommendations and to highlight similarities and differences, five key areas were selected: timing of routine visits; screening for mood disorders; maternal health checks; infant health checks; and promotion of breastfeeding. Where recommendations differed, the quality and strength of evidence cited was examined.

### Quality assessment

To objectively evaluate the quality of each guideline the Appraisal of Guidelines for Research & Evaluation Instrument (AGREE II) was used [29]. AGREE II aims to provide a uniform framework to assess the quality of guidelines, provide a methodological strategy for their development, and inform what and how information ought to be reported [30]. The instrument consists of 23 items organized into six domains: scope and purpose; stakeholder involvement; rigour of development; clarity of presentation; applicability; and editorial independence. For each item reviewers assign a score of one to seven depending on how much they agree or disagree that the guideline conforms with the provided criteria (1 = strongly disagree, 7 = strongly agree). Using a similar scale, reviewers assess the overall quality of each guideline and whether they would recommend the use of the guideline, with or without modifications.

Two authors (CH and MVD) independently scored each guideline. Domain scores were calculated by dividing the difference between the obtained score and the maximum possible score by the difference between the maximum and the minimum possible score. In keeping with similar studies, guidelines with scores of less than 50% were deemed to be of low quality [31-33].

## Results

### Selected guidelines

**Figure 1:**
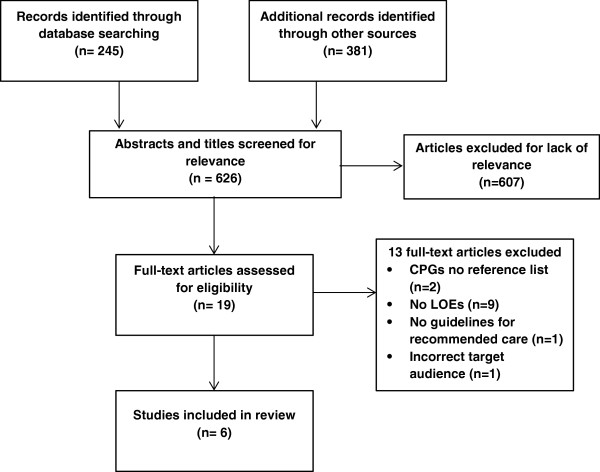
**PRISMA 2009 flow diagram outlining selection process of guidelines for analysis.** (CPGs: Clinical Practice Guidelines, LOEs: Levels of evidence).

A total of 626 references were identified by the search. The titles and abstracts of these articles were reviewed by CH and 607 references were discarded because they were not primarily clinical guidelines or their scope did not include postpartum care in primary care. The full text of a further 19 references were compared against the inclusion criteria and another 13 articles were rejected, leaving six guidelines for inclusion in the review (see Figure 1 and Table 1). These were: beyondblue ‘Depression and related disorders – anxiety, bipolar disorder and puerperal psychosis – in the perinatal period’ [34] (Australia); Faculty of Sexual and Reproductive Health (FSRH) ‘Postnatal sexual and reproductive health’ [35] (UK); Institute for Clinical Systems Improvement (ICSI) ‘Preventative services for children and adolescents’ [36] (USA); National Institute for Health and Clinical Excellence (NICE) ‘Routine postnatal care of women and their babies’ [37] (UK); Royal Australian College of General Practitioners (RACGP) ‘Guidelines for preventive activities in general practice’ [38] (Australia); and Scottish Intercollegiate Guidelines Network (SIGN) ‘Management of perinatal mood disorders’ [39] (UK).

**Table 1:** Selected guidelines – characteristics and scope

		**Beyondblue** - *Depression and related disorders – anxiety, bipolar disorder and puerperal psychosis – in the perinatal period*[34]	***Faculty of Sexual and Reproductive Health (FSRH)****– ‘Postnatal sexual and reproductive health’*[35]	***Institute for Clinical Systems Improvement (ICSI)****– ‘Preventative services for children and adolescents’*[36]	***National Institute for Health and Clinical Excellence (NICE)****– ‘Routine postnatal care of women and their babies’*[37]	***Royal Australian College of General Practitioners (RACGP) –*** *‘Guidelines for preventive activities in general practice’*[38]	***Scottish Intercollegiate Guidelines Network (SIGN)****– ‘Management of perinatal mood disorders’.*[39]
*Country of origin and year of publication*		Australia 2011	United Kingdom 2009	USA 2012	United Kingdom 2006	Australia 2012	United Kingdom 2012
*Sponsoring organisation*		Not-for-profit organization (government sponsorship)	Professional body	Government organisation	Government organisation	Professional body	Government organisation
**Maternal health**	Enquiry and assessment of physical well-being				√	√	
	Contraception		√		√		
**Maternal mental health**	Enquiry about emotional well-being	√			√	√	√
	Recommendations about screening tool	√			√	√	√
	Treatment for postnatal depression	√					√
**Infant health**	Enquiry and assessment of physical well-being			√	√	√	
	Information on healthy parent-infant relationship	√		√			
**Breastfeeding**	Recommendations promoting breastfeeding			√	√	√	
	Information regarding the use of medications during breastfeeding	√	√				√
		*Psychotropic medications only*	*Contraceptive medications only*				*Psychotropic medications only*

^√^The guideline has included this topic in the document.

The countries from where the guidelines were sourced have different maternity care systems. In Australia, women will access their GP or obstetrician/paediatrician respectively for routine care around 6 weeks. All women can also visit free Child and Infant Health Nurses regularly from birth, although not all take advantage of the service. In the USA most women will visit an obstetrician for routine care for themselves and a paediatrician for the infant, including well-baby checks. In the UK midwives play a more significant role in the birth and subsequent postpartum care. Women have access to midwifery care at home for up to a month and health visitors for the remaining postpartum period.

### Scope of the guidelines

The scope of the guidelines, under four broad themes (maternal health, maternal mental health, infant health and breastfeeding) is summarised in Table 1.

#### Maternal health

The NICE [37], RACGP [38] and Faculty of Sexual and Reproductive Health (FSRH) [35] guidelines all provided recommendations for this theme with the NICE guideline being by far the most comprehensive, covering a wide variety of topics. In contrast, the RACGP guideline had only one recommendation and the FSRH guideline focused mainly on sexual health issues and contraception.

#### Maternal mental health

Two guidelines (beyondblue [34] and Scottish Intercollegiate Guidelines Network (SIGN) [39]) focused almost entirely on maternal mental health while another two (RACGP and NICE) included recommendations for assessing women’s emotional wellbeing. The SIGN guideline discussed maternal mental health in detail under the following sub-heading: predicting and reducing risk; prevention; management; and prescribing issues and a similar level of detail was provided in the beyondblue guideline.

#### Infant health

Three of the six guidelines reviewed included a section on infant health. The NICE, RACGP and Institute for Clinical Systems Improvement (ICSI) [36] guidelines provided detailed information regarding infant physical examination. As infant health was the main focus of the ICSI guideline, it contained the most comprehensive information on this topic and included recommendations on social issues such as circumcision and second hand smoke exposure as well as oral health.

#### Breastfeeding

All six of the guidelines provided recommendations regarding breastfeeding, although for three guidelines it only related to maternal medications and the effect on breastfed infants. Areas covered included breastfeeding promotion and initiation and common problems mothers’ experience. Some of the recommendations provided by the NICE guideline applied to in-hospital care and are therefore outside the scope of this review.

### Key content areas

#### Timing of routine visits

Under its ‘clinical care pathway’ the NICE guideline key recommendations are divided into three time bands - within 24 hours, between two and seven days and between two and eight weeks (from day 8 onwards). This guideline mentions a routine six to eight week postpartum consultation and discusses some of the strengths and weaknesses of the care currently delivered in this consultation. However, this point is not listed in the overall recommendations and is graded as a ‘good practice point’ indicating it is a recommendation based on expert opinion and long-standing practice rather than direct evidence. The ICSI recommends that ‘preventive service’ visits should occur within the first two weeks after birth and at two, four, six to nine, 12 and 15 months of age. It notes, however, that there is insufficient evidence to recommend one visit schedule over another with evidence cited from two earlier guidelines with recommendations based on ‘low quality evidence’. The SIGN, beyondblue, FSRH and RACGP guidelines do not include clinical care pathways and timing of routine visits is implied rather than explicit. For example, the SIGN guideline suggests enquiring about depressive symptoms postpartum at four to six weeks and three to four months postpartum, suggesting a visit at that point in time. Similarly the beyondblue guideline recommends that screening for postpartum depression between six and 12 weeks after birth during an existing routine postpartum visit. The only statement in the RACGP guideline that includes a timeframe is that the first routine immunisation for a newborn should be at six to eight weeks.

#### Screening for mood disorders in the postpartum period

The two Australian guidelines (beyondblue and RACGP) recommend the use of the Edinburg Postnatal Depression Scale (EDPS) as a component of the assessment for depression and co-occurring depression and anxiety in postpartum women. Following a review of the existing literature (15 articles between 1987 and 2008) which found that the EPDS had a high sensitivity and specificity and therefore could be considered an appropriate screening tool, beyondblue graded their recommendation ‘B’. This means that the body of evidence can be trusted to guide practice in most situations. They recommend that screening be undertaken between six and 12 weeks postpartum.

In contrast, the NICE guideline recommends against using the EPDS as a screening tool, although it may serve as part of assessment alongside professional judgement and clinical interview. The SIGN guideline also only recommends using the EPDS or Whooley Questions (During the past month, have you often been bothered by feeling down, depressed or hopeless? During the past month, have you often been bothered by having little interest or pleasure in doing things?) to aid clinical monitoring and to facilitate discussion of emotional issues. Both guidelines recommend asking women about their emotional well-being at every visit (NICE) or at least at four to six weeks and three to four months postpartum, more frequently in women at high risk (SIGN). Both guidelines use the evidence provided in the National Institute for Health and Clinical Excellence Antenatal and Postnatal Mental Health Clinical Management and Service Guidance [40], to reference their recommendations.

#### Maternal health check

Three guidelines contained information about maternal health checks. The NICE guideline provides detailed recommendations under subheadings about routine care and specific problems women may encounter. Topics covered range from urine and bowel problems and sexual difficulties to tiredness, contraceptive use and maternal diet. Many of the key recommendations made in the clinical pathways for postpartum care were ‘good practice points’ and were based on expert opinion. The FSRH guideline focused primarily on sexual health (both physical and mental) and contraception and included recommendations about opportunistic consultations about pertinent issues and the need to be able to access appropriate information and support for women, and to refer when necessary. The RACGP guideline contained only one recommendation in this content area; that women should be asked about urinary incontinence on a yearly basis.

#### Infant health check

Three guidelines (ICSI, RACGP, NICE) contained a number of age related health checks for newborns and infants. Many recommendations involved screening for physical conditions and infant development, haemoglobinopathies, metabolic and endocrine conditions (e.g. phenylketonuria and hypothyrodisim) and other congenital problems such as hearing loss. Preventative counselling for issues including SIDS prevention/safe sleeping, non-accidental injury and immunisation is also recommended. In addition, the guidelines discuss the benefits of assessing family functioning and parent-infant interactions and providing information about available community services. The ICSI guideline recommends against the use of routine biochemical tests.

Although the recommendations provided were consistent between guidelines, the levels of evidence cited varied for the same recommendation. For example evidence for immunisation is given level A by the RACGP and Level 1 by the ICSI guideline, but only a ‘good practice point’, indicating a lack of good quality evidence supporting the recommendations, in the NICE guideline.

#### Promotion of breastfeeding

Three guidelines (RACGP, ICIS, NICE) recommend that breastfeeding should be supported and promoted regardless of the location of care. In addition, the NICE guideline provides a number of detailed recommendations including that all health care facilities should have a written breastfeeding policy that is communicated to all staff and parents. It also recommends that breastfeeding should be discussed with women at each contact with additional support being provided if necessary. Breastfeeding progress should then be assessed and documented in the postnatal care plan at each contact. Both the SIGN and beyondblue guidelines only included information on the suitability of using psychotropic drugs during the breastfeeding period while the FSRH guideline made recommendations about contraceptive methods and breastfeeding.

### Quality assessment

Five of the six guidelines were able to be reviewed using the AGREE II criteria. The RACGP guideline did not provide sufficient information for AGREE II assessment.

Overall, there was a high degree of agreement between the two independent reviewers’ AGREE II scores. This was reflected in the overall scores, with the average of all six domains for each guideline being greater than 50%. The only guideline with a domain score deemed to be low quality (less than 50%) was the editorial independence domain in the ICSI guideline. Details for the scoring for each guideline can be found in Table 2.

**Table 2:** AGREE II Domain scores of the selected guidelines (Note: the RACGP guidelines were excluded from this table as insufficient information was provided to score these guidelines using the AGREE II criteria)

	**Guideline**					
	**Beyondblue**	**FSRH**	**ICSI**	**NICE**	**SIGN**	***Domain mean***
**AGREE domain**						
Scope and purpose (%)	100	67	69	100	97	87
Stakeholder involvement (%)	97	69	56	94	92	82
Rigour of development (%)	95	54	53	79	78	72
Clarity of presentation (%)	97	92	72	89	94	89
Applicability (%)	83	50	52	65	79	66
Editorial independence (%)	75	88	33	67	54	63
***Overall mean***	91	70	56	82	82	
***Reviewers recommendations (1–7)***	7	4	4.5	7	6	

All of the guidelines received high scores for describing their scope and purpose, and stakeholder involvement. Generally the guidelines used systematic methods to select evidence and described their criteria for selection in their final documents. Most guidelines also described the methods for formulating their recommendations clearly and generally the recommendations were specific, unambiguous and easily identifiable. In comparison to other domains, most guidelines scored poorly in applicability. They neglected to include information regarding monitoring and auditing, and details regarding the implementation of recommendations into practice was rarely covered. All five guidelines failed to adequately describe how they ensured that the views of the funding body did not influence the content of the guidelines. Despite stating how the ICSI has a transparent policy with regard to disclosing conflicts of interest, actual conflicts of interest were not disclosed. Therefore this guideline was the only one to score less than 50% in any one domain.

## Discussion

Only six guidelines from Australia (2), the UK (3) and the USA (1) met the inclusion criteria and were reviewed. There was a significant variation in the scope of the guidelines with only one guideline encompassing routine postpartum care for the mother/infant dyad and providing sufficient detail to enable a practitioner to provide appropriate care to women during this period [37]. The other guidelines only focused on the infant [36], specific postpartum issues in the mother [34,35,39], or preventative activities [38].

The scarcity of comprehensive guidelines for mothers and infants is a concern because of the stress many women experience at this time, the high burden of maternal morbidity postpartum [41] and the significant interplay between the health of the mother and infant. However, developing wide-ranging, high quality guidelines is a time and resource intensive activity and this may be one reason for so few well researched guidelines. In addition, this finding may be a reflection of a lack of high-quality research into the most effective care for postpartum women and their infants in the community, especially as the level of evidence for many of the recommendations in the NICE guidelines was ‘good practice points’.

The lack of evidence is especially apparent when considering the recommendations for the timing of routine visits. While two guidelines had explicit timing for visits (although they were not consistent), the others implied health professional contact at various times throughout the postpartum period. The ISCI guideline explicitly states that there is no evidence that one regime for postpartum visits is better than another [36]. Other authors also raise questions about the most appropriate timing of postpartum visits to support, encourage and reassure women and to prevent, identify and manage issues that may arise [42-44]. Considering a more mother-centred approach to the timing of visits, rather than having an all-inclusive recommendation, may be appropriate. This would require clear advice to the mother about when to seek assistance and to the practitioner about the time frame for a visit depending on whether all was going well or not.

In addition, although three guidelines discuss performing routine examinations on infants, the ISCI again states that there is no evidence that examining an asymptomatic child is beneficial in identifying occult disease [36]. However, these examinations may provide reassurance and an opportunity to deliver anticipatory guidance to the mother.

There was also inconsistency across guidelines in regards to the screening of women for postpartum depression with two recommending and two not recommending the use of EPDS. The recommendations in all four guidelines are the result of systematic reviews of the literature. However, the review conducted by beyondblue (on which the RACGP recommendation is based) was conducted at later point in time than that used by NICE and SIGN when additional high quality trials of the use of the tool had been undertaken. These findings stress the need to update guidelines on a regular basis as new evidence is accumulated [45].

Variability between guideline recommendations is not uncommon [46-48]. Matthys et al. reports that differences in recommendations could be due to ‘*insufficient evidence, different interpretations of the evidence, unsystematic guideline development methods, the influence of professional bodies, patient preferences, cultural and socioeconomic factors or characteristics of the health care system*’ [46]. A number of these issues may have had an impact on the variability seen with the guidelines in this review. Having a strict process for guideline development will go some way to producing more consistent and high quality guidelines. It is also appropriate to have evidence-based guidelines that reflect the cultural and health care systems in a particular region [49,50].

The AGREE II instrument can assist guideline developers as well as providing a framework for reviewing quality of guidelines, as was undertaken in this review. Despite all six guidelines being published after the release of the AGREE instrument, only one guideline (RACGP), mentioned its use during guideline development. Surprisingly, the RACGP guideline failed to report their methodology sufficiently for reviewers to use the AGREE II criteria to score the guideline. As noted by Greuter et al. [32], one would assume that guideline developers are aware of the AGREE instrument and up-to-date with the literature about reporting the methodology of guideline development [32]. The possibility that guidelines of high methodological quality score poorly on the AGREE II instrument due to failed reporting, highlights the need for authors to consider and report quality measures when developing the guidelines.

Similar to prior reviews of guidelines, applicability and editorial independence domains scored the lowest [31,32]. In the case of editorial independence, low scores were mostly assigned due to authors failing to adequately report their methodology. Given that implementation is the key objective of most guidelines there needs to be an improvement in the applicability domain in future revisions of guidelines. It was also concerning to see the FSRH guideline failing to provide a procedure for updating its recommendations considering the importance of guidelines remaining up-to-date.

### Limitations

One limitation of the study was the exclusion of guidelines that were not available to download over the internet. However, guidelines are there to guide clinical practice and need to be readily available to those who need them. Another limitation was the inclusion of English language guidelines only. This may have led to the exclusion of other relevant guidelines, designed for use in areas such as continental Europe and Asia that may have brought a different cultural perspective to the review. Although attempts were made to obtain all guidelines that fitted the inclusion criteria, some may have been missed if they were not retrieved with the search terms used.

## Conclusion

Clinical practice guidelines provide an avenue for practitioners to access critically-evaluated evidence-based recommendations for the care of their patients. This review found only six guidelines from the international literature that satisfied the selection criteria and addressed out-of-hospital maternal and infant care in the postpartum period. Despite the quality of the guidelines and the similarity of recommendations, only one guideline covers routine postpartum care for the mother and infant. It is important that the mother and infant be seen as a unit, particularly in the first few months of life, because what affects one inevitably affects the other. It is also important not to position normal postpartum care within an illness framework when most women and infants have an uneventful course. Further research into care within the postpartum period is warranted as many recommendations, such as timing of visits and maternal and infant examinations, are not backed up by high levels of evidence, often relying on historical models of care and the status quo. In addition, there needs to be increased rigor into formulating guidelines, or at least in reporting the development of guidelines – especially with regard to editorial independence and mechanisms to up-date the guidelines.

## Abbreviations

AAP: American Academy of Pediatrics; AGREE ll: Appraisal of guidelines for Rrsearch & evaluation instrument; EDPS: Edinburg postnatal depression scale; ICSI: Institute for clinical systems improvement; NGC: National guideline clearinghouse; NICE: National Institute for Health and Care Excellence; RACGP: Royal Australian College of General Practitioners; ROCG: Royal College of Obstetricians and Gynaecologists; SIGN: Scottish Intercollegiate Guidelines Network; UK: United Kingdom; USA: United States of America.

## Competing interests

The authors have no competing interests.

## Authors’ contributions

CH participated in the design of the study, undertook the literature search, summarised the guidelines, assessed the guidelines for quality and wrote the majority of first draft of the manuscript. MVD conceived and participated in the design of the study, reviewed the literature search and guideline summaries, assessed the guidelines for quality and reviewed and revised the manuscript. BM conceived and participated in the design of the study, reviewed the literature search, guideline summaries and quality assessment and reviewed and revised the manuscript. WB conceived and participated in the design of the study, reviewed the literature search, guideline summaries and quality assessment, contributed to the first draft of the manuscript and edited subsequent drafts. All authors read and approved the final manuscript.

## Pre-publication history

The pre-publication history for this paper can be accessed here:

http://www.biomedcentral.com/1471-2393/14/51/prepub
